# Engineering Myocardium for Heart Regeneration—Advancements, Considerations, and Future Directions

**DOI:** 10.3389/fcvm.2020.586261

**Published:** 2020-10-15

**Authors:** Dillon K. Jarrell, Ethan J. Vanderslice, Mitchell C. VeDepo, Jeffrey G. Jacot

**Affiliations:** ^1^Jacot Laboratory for Pediatric Regenerative Medicine, Department of Bioengineering, University of Colorado Anschutz Medical Campus, Aurora, CO, United States; ^2^Department of Pediatrics, University of Colorado Anschutz Medical Campus, Aurora, CO, United States

**Keywords:** tissue engineering, heart disease, cardiac patch, vascularization, immune system, tissue architecture, heart defects, pluripotent stem cells

## Abstract

Heart disease is the leading cause of death in the United States among both adults and infants. In adults, 5-year survival after a heart attack is <60%, and congenital heart defects are the top killer of liveborn infants. Problematically, the regenerative capacity of the heart is extremely limited, even in newborns. Furthermore, suitable donor hearts for transplant cannot meet the demand and require recipients to use immunosuppressants for life. Tissue engineered myocardium has the potential to replace dead or fibrotic heart tissue in adults and could also be used to permanently repair congenital heart defects in infants. In addition, engineering functional myocardium could facilitate the development of a whole bioartificial heart. Here, we review and compare *in vitro* and *in situ* myocardial tissue engineering strategies. In the context of this comparison, we consider three challenges that must be addressed in the engineering of myocardial tissue: recapitulation of myocardial architecture, vascularization of the tissue, and modulation of the immune system. In addition to reviewing and analyzing current progress, we recommend specific strategies for the generation of tissue engineered myocardial patches for heart regeneration and repair.

## Introduction

Heart disease is the leading cause of death in the United States (1). Each year, nearly one million Americans suffer myocardial infarctions (MI) that are often fatal and at best lead to necrotic regions of myocardium that cause pathologic remodeling and arrhythmias. Among infants, congenital heart defects (CHD) are the most common cause of death and often require reconstructive surgeries involving the implant of inert patches in the place of myocardium (2, 3). Because the heart has almost no capacity for self-renewal, there is a clinical need for regenerative therapies that can drive myocardial regeneration in MI patients and be used for reconstructive surgeries in CHD patients. Numerous studies of scaffold-free therapies involving the direct delivery of cells, extracellular matrix (ECM), drugs, or biologics for MI patients show promise (4–6). This review, however, will focus predominantly on progress toward a tissue engineered myocardial patch (TEMP) that could be sutured over myocardial infarct regions or full wall-thickness CHD, such as septal defects. To engineer clinically useful tissue for any organ, three principal considerations must be addressed: the architecture of the tissue, vascularization of the engineered tissue, and the host immune response after implant.

The architecture of tissue—the composition and arrangement of its ECM and various cell types—is critically important to the function of all tissue types. As a whole, the heart is designed as an elegant and intricate system of valves, conduits, and pumping chambers that drives blood circulation throughout the body. The muscular heart walls are composed of a thick myocardial layer sandwiched between two single-cell layers called the endocardium (blood-contacting surface) and the epicardium (outer layer of heart). Both the epicardium and endocardium are essential for proper myocardial development, growth, and remodeling (7, 8). In the ventricular myocardium, overlaying layers of cardiomyocytes are oriented in left-handed, circumferential, or right-handed directions, resulting in an efficient torsional “wringing” motion of contraction (9, 10). ECM components include predominantly collagens, but also fibrillins, fibronectin, laminin, and periostin (11). The precise composition, fiber orientation, and crosslinking of the cardiac ECM regulates tissue strength, heart stiffness, cell proliferation, pumping efficiency, and fibroblast behavior (12–18).

**Figure 1:**
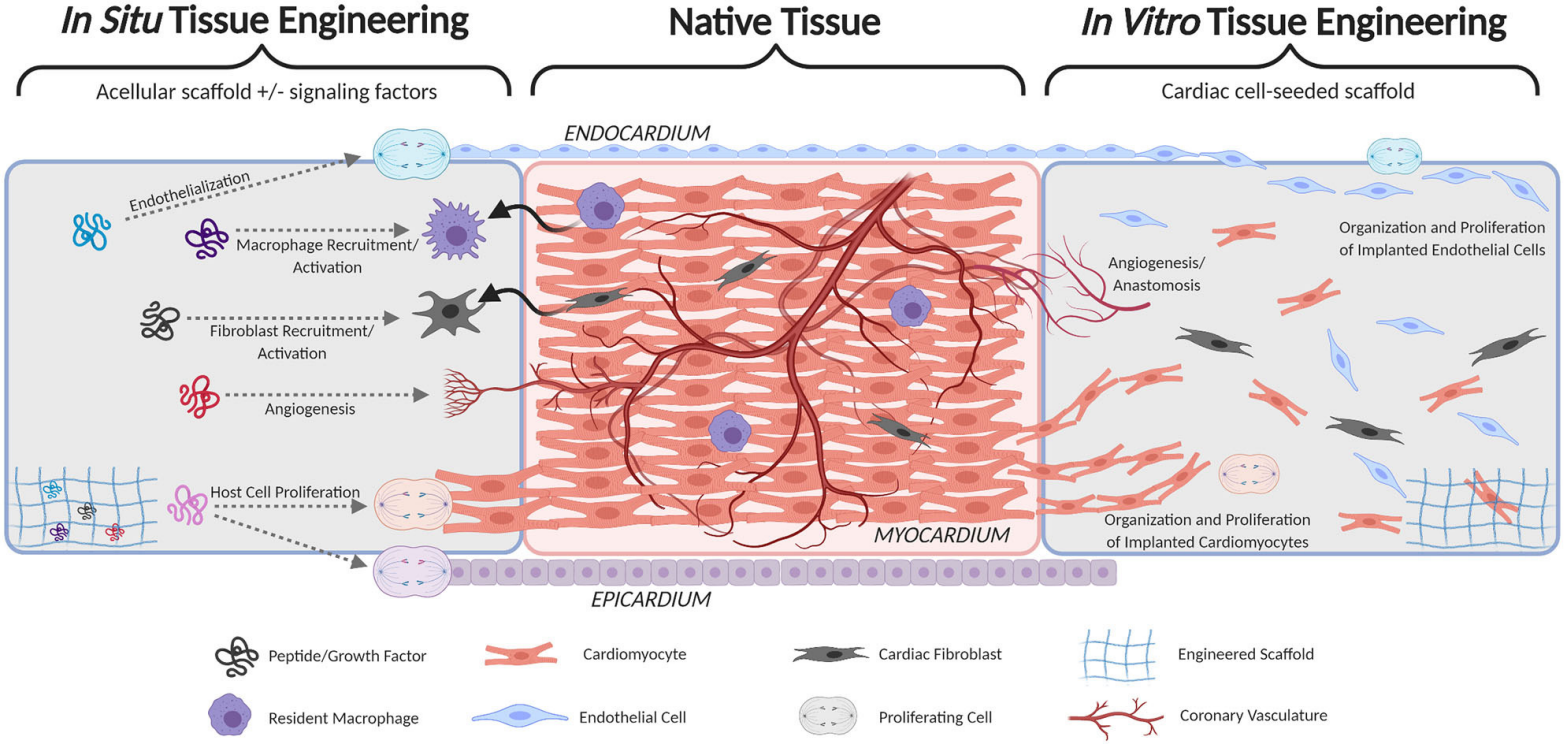
Schematic comparison of cardiac patches implementing *in situ* cardiac tissue engineering (left) and *in vitro* cardiac tissue engineering (right). Both seek to fully integrate with native host tissue (middle) by forming continuous endocardium, myocardium, epicardium, and vasculature.

In addition to cardiomyocytes, the myocardium is innervated, heavily vascularized, and populated by resident macrophages and cardiac fibroblasts (Figure 1) (19, 20). Vascularization sustains the highly metabolic muscle and allows for muscle hypertrophy during development. A method of nutrient supply is required to generate TEMPs with physiologic cell densities, and rapid anastomosis with host vasculature must be achieved shortly after implant. Within native myocardium, resident macrophages regulate the inflammatory response and are highly active following injuries, such as MI. The host immune response must be considered in TEMPs in avoiding immune rejection and may also be leveraged to improve regenerative therapies. Promoting cardiomyocyte regeneration and connectivity remains a primary hurdle in cardiac repair following malformation or injury. The architecture, vascular supply, and immune response are reviewed here as three primary approaches to coordinate regeneration and integration of engineered cardiac tissue.

## Recapitulating the Architecture of the Heart Wall

In engineering this complex myocardial tissue, the necessary degree of architectural recapitulation is unknown. The *in situ* tissue engineering approach aims to provide a remodelable scaffold and to harness the natural healing response (inflammation, recellularization, and remodeling) to achieve native tissue architecture and function (Figure 1). Numerous natural (e.g., collagen, fibrin, and native ECM) and synthetic [e.g., poly(glycerol sebacate), polyurethane, and polyethylene glycol] scaffold materials have been tested for myocardial regeneration. These materials enable fine-tuning of various important tissue properties including degradation kinetics, elastic modulus, porosity, and immune response (21–30). In addition, several studies have sought to identify or integrate various signaling factors that can modulate cardiac healing—especially cardiomyocyte proliferation—including growth factors (e.g., NRG-1, FGFs, VEGF, IGF-1), ECM components (e.g., Agrin, Fibronectin, Periostin), and modulators of the Wnt and Hippo pathways (31–36). The *in situ* approach is attractive because of its simplicity and relative ease of translation (manufacture, storage, handling, etc.), however the regenerative potential of the heart is notoriously limited. Thus, far, no combination of signaling factors and/or ECM components has been shown to drive significant myocardial regeneration. Therefore, a scaffold alone, even if loaded with signaling factors, is unlikely to drive sufficient cardiomyocyte proliferation and migration necessary for total regeneration.

Alternatively, the *in vitro* tissue engineering approach seeks to create living heart tissue prior to implant by combining a scaffold with cardiomyocytes (Figure 1). Induced pluripotent stem cell-derived cardiomyocytes (iPSC-CM) are the most promising source of cardiomyocytes for tissue engineered myocardium because they can be patient-specific, generated in large quantities, and are relatively proliferative compared to native cardiomyocytes (37, 38). Several versions of engineered myocardium using 3D scaffolds and iPSC-CM have been developed in the past decade (39–51), and these studies unanimously demonstrate that 3D culture of iPSC-CM improves their maturation and connectivity. Combining iPSC-CM with other cardiac cell types in 3D scaffolds has further improved tissue function. In particular, the inclusion of cardiac fibroblasts in tissue engineered myocardium is now considered a near-necessity because of their recently-discovered importance in the viability, organization, durability, and function of engineered and native myocardium (52–55). Coculture with endothelial cells has also been shown to improve iPSC-CM maturity, function, and proliferation and is discussed further in the following section in the context of engineering vasculature (56–58).

Homogenous co-culture of iPSC-CM with other cell types yields clear benefits, but none of these patch designs have sought to recapitulate the trilayer organization (endocardium, myocardium, epicardium) of heart muscle despite the critical signaling roles that these cell layers play on cardiac fibroblast behavior, cardiomyocyte proliferation, and tissue organization. This is likely due to the longstanding difficulty of attaining endocardial and epicardial cells. However, the increasing number of protocols for the differentiation of iPSC-derived endocardial (59) and epicardial (60) cells may open the door for studies investigating their inclusion in tissue engineered myocardium. Recapitulation of this architecture through inclusion of an engineered endocardium and epicardium using iPSC-derived cells and a trilayer scaffold could significantly improve the viability, integration, and growth of implanted TEMPs. Multi-layer scaffolds have been developed and investigated for several soft and hard tissue engineering applications using various fabrication techniques (25, 49, 61–71). Similar approaches could be employed to generate endocardium-myocardium-epicardium tissues *in vitro*. Such a trilayer tissue would also provide a useful *in vitro* model for studying aspects of heart development involving cell-layer crosstalk, including myocardial trabeculation and thickening, valve formation, and the epithelial-mesenchymal transition (EMT) of cardiac fibroblasts derived from these two cell layers.

## Vascularization of Cardiac Patches *in vitro* and *in situ*

Incorporation of vascular cell types into 3D cardiac tissues increases the proliferation and maturation of iPSC-CM and accelerates needed vascularization after implant, resulting in more clinically relevant *in vitro* tissues. While electrical and mechanical conditioning can also drive iPSC-CM maturation, 3D co-culture with endothelial cells (EC) and stromal cells appears vital in maximizing the functional maturity of iPSC-CM. Early attempts at vascularized cardiac tissues showed that in porous polymer scaffolds, EC combined with embryonic fibroblasts formed networks that did not disrupt the contractility of embryonic stem cell-derived cardiomyocytes. Additionally, the presence of EC increased the proliferation of immature cardiomyocytes and increased the maturation of non-dividing cardiomyocytes (72). Over time, further evidence of the importance of vascular cell types in 3D cardiac tissues has been increasing. EC and smooth muscle cells increased the expression of electrical and mechanical junctions in cardiomyocytes in a fibrin-based hydrogel and helped show that non-myocytes are necessary for the electrical coordination of cardiomyocytes in 3D structures (40). In collagen matrices, addition of EC enhanced the proliferation of cardiomyocytes undergoing stress conditioning (73). Bilayer constructs of cardiomyocytes in a fibrin matrix layered on a microvessel fibrin matrix containing EC and pericytes improved survival, maturation, and twitch force production of the cardiomyocytes compared to constructs without the microvessel layer (43). Furthermore, multiple groups have assessed the drug response of 3D multicell culture systems, unanimously demonstrating that vascularized 3D tissues have significantly different toxicity responses compared to 2D and cardiomyocyte-only systems. These differences have been attributed primarily to more mature contractile properties and calcium handling (74–77). The mechanisms of cellular cross-talk that lead to CM maturation continue to be investigated (78), but it is clear that vascular cell types in 3D culture systems significantly improve CM maturation and function. With evidence of the importance of vascular cell types in cardiac constructs, optimizing the ratio of iPSC-CM to vascular cells has become of interest. Different ratios have been shown to lead to different levels of cardiomyocyte function and maturity (79, 80). However, the optimal ratio may depend on the overall goal and approach to 3D culture (e.g., drug screening vs. therapeutic implant, scaffold-free vs. cell sheet layering) as the degree of vasculature is especially important for *in vivo* survival.

In addition to improving proliferation and maturation of cardiomyocytes *in vitro*, vascularization is necessary for creating larger cardiac constructs that survive and engraft *in vivo*. An *in situ* approach of releasing angiogenic growth factors from CM patches has shown to increase vessel ingrowth and improve overall outcomes in rodent myocardial infarction models compared to non-angiogenic controls (81, 82). However, the *in vitro* approach that adds vascular cells to living cardiac constructs may provide quicker and more robust vascular development for MI repair and will be necessary for developing reconstructive tissues for CHD repair. Incorporation of vascular cells into cardiomyocyte constructs led to >10-fold larger cell grafts than cardiomyocyte-only constructs, which became necrotic upon implantation on skeletal muscle (83). Vascular cells in 3D constructs increase the ingrowth of host vasculature and form vessels that can anastomose to the native vasculature and become perfusable (43, 58, 73, 84). The rapid formation of perfusable vasculature in cardiac implants is necessary for survival and function of the implanted iPSC-CM. Recent analysis showed that encapsulated vascular cells that were allowed to self-assemble into networks did not integrate as quickly or to the same degree as vascular cells that were seeded into pre-formed perfusable channels when grafts were implanted onto rat hearts. Consequently, the perfusable vascular grafts showed higher cardiomyocyte and vascular densities within the implant (85). It is important to note that the majority of vascularized cardiac patch research so far has been in rodent models, which have significantly thinner myocardium than larger animals and humans. Scaling up the size of pre-vascularized constructs remains challenging. The number of layered cell sheets of cardiomyocytes and ECs has a limit before necrosis occurs (86). Thus, it appears that engineering scaffolds with perfusable channels or vessels instead of relying on self-assembled vascular networks will be necessary for larger constructs. Potential approaches to robust vasculature in larger tissues have been explored, including decellularizing and then recellularizing pig ventricles connected to a perfusion bioreactor (87), 3D printing vascularized constructs (88), and the AngioChip which can be surgically anastamosed to host vasculature (89).

## Harnessing the Immune System in Cardiac Tissue Engineering

The role of the immune system in cardiovascular tissue engineering is becoming increasingly apparent. Research on regenerative therapies and tissue engineering solutions for cardiac tissues has significantly advanced our understanding and ability to modulate both the negative inflammatory response and encourage a tissue repair response through immune pathways. Following myocardial injury, the inflammatory response is critical in helping protect the necrotic infarct area by increasing stiffness and preventing infarct expansion; however, inflammation may also lead to adverse remodeling of the ventricular wall leading to heart failure. Recruitment of inflammatory cells to the site of inflammation occurs through chemokine paracrine signaling, notably via the CCL2 (c-c chemokine ligand 2; MCP-1) and CX3CL1 [chemokine (C-X3-C motif) ligand 1] cytokines and their associated receptors, CCR2 and CX3CR1 (90). Following injury to the myocardium, resident macrophages aid in recruiting CCR2+ macrophages from the bloodstream, which display increased pro-inflammatory (M1) and anti-inflammatory (M2) markers (91). Inhibition of the CCR2 protein resulted in decreased inflammation following MI in mice and decreased adverse remodeling of the ventricular wall, indicating the recruited cells may be responsible for adverse remodeling (92–94).

However, the infiltration of inflammatory cells is not always an adverse response, and in fact may be leveraged to enhance tissue-engineering strategies. The majority of research into this space has been tested using direct injection of cells into infract zones. It was originally believed cell therapy acts via integration of seeded cells, though it is now thought to act through paracrine signaling and recruitment of autologous cells (95). The inflammatory response is critically linked to this process as demonstrated by an observed reduction in heart regeneration when macrophages are depleted pre-injury (96). Recent work into the mechanisms provides greater insight. Mononuclear cells or the immune activator zymosan injected into a mouse heart post-MI was shown to increase recruitment of CCR2+ and CX3CR1+ monocytes, which increased heart function (90). Other approaches have aimed at directly modulating the immune response with cytokines. Pre-treating MSCs with the pro-inflammatory cytokine TNFα prior to injection in an MI rat model led to increased recovery of cardiac function (97). Further work using genetic knockouts found this cardiac protection acts via the TNFR2 receptor, while the TNFR1 receptor does not aid in cardiac protection (98). Other cytokines tested for modulating the immune response include interleukin-10 (IL-10), SDF1a, and RANTES (99–103).

While much of the work in modulating the inflammatory response has focused on injectable approaches, the inflammatory response is equally important in the context of TEMPs. The structure, pore size, material composition, and surface topology of scaffold materials affect the host inflammatory response by modulating cell recruitment, infiltration, and activation (104). The source of seeded cells also influences the inflammatory response. Autologous cell sources are preferred, however time-consuming cell culture is required for reprogramming and differentiation. On the other hand, an off-the-shelf cell source requires HLA type matching. Thus, there is interest in generating HLA-null master iPSCs as a hypo-immunogenic cell source (105). Aside from scaffold and cell considerations, there has been very little work in modulating the immune response with a myocardial patch despite the obvious role it plays in both cardiac injury and healing (106). The MAGNUM clinical trial was an early attempt at a myocardial patch seeded with bone marrow mononuclear cells and demonstrated slight success, likely acting through inflammatory pathways (107). There is great potential in modulating the immune response, both in limiting an adverse response and promoting a healing response, and future work in cardiac tissue engineering should consider leveraging these pathways to enhance outcomes. Specifically, the inclusion of immunomodulatory molecules within a TEMP could be used to positively direct the inflammatory response. For example, since the temporal availability of injected cytokines is short, hydrogels of varying degradation rates have been tested to provide a time-controlled release of Met-CCL5 and CXCL12. These hydrogels led to decreased early neutrophil infiltration, increased neovascularization, and decreased apoptosis in the infarcted myocardium (101). Finally, it should be noted that heart disease patients are not in perfect health, so the pre-existing inflammatory state of the heart must be considered. For example, atherosclerosis, the leading cause of MI, is modulated via inflammatory pathways, specifically CCL2 (108). This leads to chronic inflammation in the surrounding tissues such that following MI, the recruited macrophages are infiltrating a site of chronic inflammation, altering their response and delaying or preventing a regenerative phase (109). Such considerations are often not taken into account for many of the animal models used to test MI therapies, as the injury is often acutely simulated and no pre-existing inflammatory state exists. Cardiac tissue engineering, either via the *in situ* or *in vitro* approach, must consider the pre-existing inflamed status of ischemic myocardium as it may alter the regenerative outcome of the implanted biomaterial.

## Conclusions

The prevalence of heart disease throughout the human population and the limited supply of donor hearts has created a need for TEMPs that could be used to regenerate myocardial infarct regions or in reconstructive surgeries in CHD patients. We think that an *in situ* (acellular) tissue engineering strategy would require a suturable, biodegradable scaffold loaded with angiogenic and immunomodulatory signaling factors that encourage both angiogenesis and a healing immune response. However, given the extremely low proliferation rate of cardiomyocytes, we believe that an *in vitro* (cellularized) tissue engineering approach is the most promising. We think that such a TEMP requires at minimum a biodegradable scaffold seeded with either (1) a low density of proliferative iPSC-CM, cardiac fibroblasts, and EC (that can survive by simple media/blood diffusion prior to the development of a proper vasculature) or (2) a high, more-physiologic density of these cell types in a scaffold that includes engineered vascular channels that can provide perfusion *in vitro* and rapid anastomosis *in vivo*. Recapitulation of cardiac innervation in engineered patches could be necessary for complete patch integration *in vivo*, however action potentials in the myocardium are primarily transmitted through the cardiomyocyte syncytium (110). Therefore, promoting cardiomyocyte regeneration and connectivity is likely to be more important in cardiac tissue engineering than the inclusion of neural networks.

In addition to these minimum requirements of an *in vitro* TEMP, we postulate that a more complex TEMP that also recapitulates the endocardium and epicardium is the most promising approach for heart regeneration. With any of these strategies, the rate of TEMP integration in the host heart needs to exceed the degradation rate of the scaffold to prevent patch rupture during remodeling. In addition, the scaffold and its degradation products need to be tolerated by the host immune system but should also be considered an immunomodulatory tool that can be leveraged for improved healing and integration. Maintaining the viability of implanted iPSC-cardiomyocytes also remains a challenge that must be addressed prior to clinical translation. The inclusion of an endocardium in the patch, optimizing the maturation level of the cardiomyocytes, and modulating the host immune response are all potential strategies for increasing iPSC-CM viability and electrical integration after implantation.

Tissue engineered myocardium represents an important step toward the cure of heart disease and would also be a significant milestone as the field moves toward the eventual engineering of a whole bioartificial heart. Furthermore, the considerations, challenges, and advancements reviewed herein are translatable to many different tissue engineering endeavors, but especially to other cardiovascular applications including tissue engineered heart valves and large vessels.

## Author Contributions

DJ, EV, MV, and JJ contributed equally to conceptualization of the work. DJ was responsible for manuscript organization, figure development, and primary authorship. EV was the lead author on section Vascularization of Cardiac Patches *in vitro* and *in situ*. MV was the lead author on section Harnessing the Immune System in Cardiac Tissue Engineering. All authors contributed to the article and approved the submitted version.

## Conflict of Interest

The authors declare that the research was conducted in the absence of any commercial or financial relationships that could be construed as a potential conflict of interest.
